# Vergleichende Analyse der Refraktions- und Topographieveränderungen nach lidchirurgischen Eingriffen

**DOI:** 10.1007/s00347-021-01361-0

**Published:** 2021-03-18

**Authors:** J. Mehlan, B. Jonca, S. Dulz, S. Green, M. S. Spitzer, F. Schüttauf

**Affiliations:** Klinik und Poliklinik für Augenheilkunde, Universitätsklinikum Hamburg Eppendorf, Martinistr. 52, 20246 Hamburg, Deutschland

**Keywords:** Lidchirurgie, Blepharoplastik, Levatorresektion, Hornhauttopographie, Astigmatismus, Eyelid surgery, Blepharoplasty, Levator resection, Corneal topography, Astigmatism

## Abstract

**Hintergrund und Ziel der Arbeit:**

Über die Frage, ob nach Blepharoplastik, Levatorresektion oder lateraler Zügelplastik Refraktions- oder Topographieveränderungen zu erwarten sind, besteht weitestgehend Unklarheit.

**Material und Methoden:**

Daher wurden in der vorliegenden Studie prä- sowie postoperativ von 78 Patienten objektive Refraktion, Vorderabschnittstomographie mittels Pentacam und Visusprüfung durchgeführt und der Analyse zugeführt. Die Untersuchung erfolgte präoperativ, zum Zeitpunkt des Fadenzugs nach 10 Tagen sowie nach 3 Monaten.

**Ergebnisse:**

Wir fanden postoperativ weder nach Blepharoplastik noch nach lateraler Zügelplastik signifikante Veränderungen von Visus oder Refraktion noch in der Topographie. Hingegen zeigt der Wilcoxon-Vorzeichen Test 10 Tage nach der Levatorresektion einen signifikanten Anstieg des Zylinders nach 10 Tagen im Vergleich zum präoperativen Niveau (*p* = 0,042). Diese Veränderung war jedoch nach 3 Monaten nicht mehr nachweisbar.

**Schlussfolgerung:**

Wir postulieren daher, dass eine umfangreiche Aufklärung von Patienten hinsichtlich passagerer Sehveränderungen insbesondere bei Levatorresektionen unabdingbar ist und postoperativ eine ergänzende Refraktions- und Topographiekontrolle sinnvoll sein kann.

## Abstract (en)

**Background:**

There is largely a lack of clarity on the question of whether refraction or topography changes are to be expected after blepharoplasty, levator resection, or a lateral tarsal strip procedure.

**Materials and methods:**

Therefore, in the present study, objective refraction, anterior eye segment tomography, and visual acuity tests were carried out pre- and postoperatively in 78 patients and then analyzed. The examination was carried out preoperatively, at suture removal after 10 days, and after 3 months.

**Results:**

Neither after blepharoplasty nor after a lateral tarsal strip procedure were significant changes in vision or refraction seen in the topography. In contrast, the Wilcoxon sign test 10 days after levator resection showed a significant increase in the cylinder after 10 days compared to the preoperative level (*p* = 0.042). However, this change was no longer detectable after 3 months.

**Conclusion:**

The authors postulate that extensive patient education with regard to temporary visual changes, particularly in the case of levator resections, is essential and that additional refraction and topography control can be useful postoperatively.

Fehlstellungen der Augenlider, z. B. Ptosis oder Blepharochalasis, können die corneale Oberfläche modifizieren und ggf. auch refraktive Fehler induzieren [4].

Zur Korrektur einer Lidfehlstellung werden je nach zugrunde liegender Pathologie verschiedene Operationsverfahren eingesetzt.

Jedoch kann nicht nur die Lidfehlstellung die corneale Oberfläche und damit auch die Refraktion verändern. Auch die operative Korrektur und damit veränderte Auflage und Zugrichtung der Lider kann Ursache solcher Veränderungen sein.

Im Zuge unserer Studie werden die Oberlidblephroplastik zur Korrektur einer Dermatochalasis, die Levatorresektion zur Korrektur der Ptosis und die laterale Zügelplastik zur Korrektur von Unterlidfehlstellungen wie beispielsweise En- oder Ektropium betrachtet.

Ziel unserer Untersuchungen war die Analyse, ob es durch die operative Korrektur der Lidfehlstellung zu Veränderungen von Refraktion, Visus oder cornealer Topographie kommt.

## Methodik

Im Studienzeitraum von September 2017 bis Mai 2019 wurden 2000 Patienten lidchirurgisch operiert. Hierunter fallen 184 alleinige Blepharoplastiken, 111 Levatorresektionen und 72 solitäre laterale Zügelplastiken. Ausgenommen wurden von dieser Evaluation kombinierte Operationen oder auch Operationen, bei denen die genannten Eingriffe jeweils nur einen Teilaspekt behandelten. Diese Operationen stellten aber einen Großteil der 2000 Eingriffe dar.

Alle Eingriffe wurden von vier erfahrenen Operateuren in gleicher Technik durchgeführt. Einzige Ausnahme stellt die Verwendung des CO_2_-Lasers für die Schnittführung bei der Levatorresektion und Blepharoplastik dar. Dieser wurde von einer Kollegin verwendet.

Eingeschlossen wurden Patienten über 18 Jahre mit rein medizinischer Indikation zur Korrektur der Lidfehlstellung. Es wurden nur Patienten eingeschlossen, die keine ophthalmologischen Vorerkrankungen hatten und zur ersten Operation der Lider kamen. So sollten möglichst viele Bias durch z. B. vorexistierende Narben, stärkere Schwellung bei Reoperationen ausgeschlossen werden.

Es erfüllten 120 Patienten die Einschlusskriterien und willigten ein, an der prospektiven Studie teilzunehmen.

78 Patienten absolvierten das Follow-up vollständig. Lediglich die vollständigen Datensätze wurden für die Auswertung verwendet.

Das Vorhaben wurde durch die örtliche Ethikkommission beraten und genehmigt.

### Ablauf

Nach vorangegangener Information und Aufklärung wurden die Patienten präoperativ sowie postoperativ nach zehn Tagen zum Termin des Fadenzugs und nach 3 Monaten mittels objektiver Refraktion (*NIDEK-ARK-560A, Oculus, Wetzlar*) und Pentacam (*Pentacam®, Oculus, Wetzlar) *untersucht. Zusätzlich erfolgte zu jedem der 3 Zeitpunkte eine Visusprüfung.

Die Gruppierung erfolgte entsprechend der durchgeführten Operation (Gruppe 1: Blepharoplastik, Gruppe 2: Levatorresektion, Gruppe 3: laterale Zügelplastik).

#### Operative Prozeduren

##### Oberlidblepharoplastik.

Zunächst wurde die Schnittführung eingezeichnet. Die mediale Grenze stellt das obere Tränenpünktchen dar. Die Schnittführung wird in die Deckfalte des Oberlids gelegt. Die obere Grenze sollte nicht dichter als 10 mm bis an die Braue heran reichen. Als laterale Begrenzung ist die Orbitakante zu sehen. Sollte es weiter medial und lateral noch deutliche Hautüberschüsse geben, so wären zusätzliche Hautausschneidungen (als Burow-Dreiecke) zu erwägen.

Der so eingezeichnete Hausüberschuss wird exzidiert, und nach bipolarer Blutstillung erfolgt die Hautnaht.

##### Levatorresektion.

Nach erfolgter Lokalanästhesie führten wir eine Hausinzision mit dem Skalpell gemäß der beschriebenen Schnittführung durch. Durch Anheben des Wundrands wird eine stumpfe Präparation auf die Vorderfläche des Tarsus möglich, und auch oberhalb der Tarsalplatte wurde stumpf auf das Septum präpariert. Nachdem das präaponeurotische Fett sichtbar wird, kann das Septum eröffnet werden [3].

Die Aponeurose wurde vom Unterrand des Tarsus abgetrennt, und über der Pupille wird mittels einer ersten selbstresorbierenden Naht an der Vorderfläche des Tarsus refixiert. Die Kontur des Oberlids wird über die aktive Lidöffnung kontrolliert und kann durch zumeist zwei weitere Nähte medial und lateral der zentralen Naht formiert werden. Überstehende Anteile der Aponeurose werden gekürzt, und es erfolgt der Wunderschluss mit zusätzlichen lidfurchenbildenden Nähten.

##### Laterale Zügelplastik.

In unserer Klinik wurde die laterale Zügelplastik nach dem klassischen Ansatz nach Collins durchgeführt. Nach einer Inzision im Bereich des lateralen Kanthus (ca. 1–1,5 cm) erfolgte eine weitestgehend stumpfe Präparation bis zum knöchernen Rand der Orbitakante. Die Tarsalzunge wird gebildet, indem der inferiore Anteil des lateralen Lidbändchens eingeschnitten wurde. Von den so entstehenden Zügeln wird die Lidhaut entfernt, und auch die Konjunktiva wird entfernt (mit dem Skalpell). Durch eine doppelt vorgelegte 4‑0-Mersilene-Naht wird der Zügel angeschlungen und an der inneren Orbitakante, ca. 1–2 mm oberhalb des Höhenniveaus des medialen Lidbändchens, fixiert. Der Knoten wird submuskulär versenkt, und es erfolgt ein zweischichtiger Windverschluss (Musculus orbicularis und Haut) [1, 2].

### Statistik

Das Ziel der Untersuchungen war es, die Veränderung der Variablen über 3 Zeitpunkte hinweg zu testen. Da die Verteilungen der Differenzen nicht die erforderlichen Annahmen einer parametrischen ANOVA für wiederholende Messungen erfüllten, wurde ANOVA für gestutzte Mittelwerte verwendet [11].

Dabei wird die folgende Hypothese getestet: H0: μt1 = μt2 = μt3. Mit anderen Worten, diese Hypothese prüft, ob die winsorisierten Mittelwerte in 3 Zeitintervallen gleich sind.

Als Post-hoc-Test wurde der Yuen-Test für gestutzte Mittel verwendet [11]. Zusätzlich wurden sowohl der klassische T‑Test als auch der Wilcoxon-Vorzeichen-Test berechnet. Alle Daten wurden in Boxplots visualisiert und die Analysen mit R Development Core Team (2008) durchgeführt.

Zur Analyse des chirurgisch induzierten Astigmatismus wurde die Analyse nach Alpins eingesetzt [12].

## Ergebnisse

Im hier durchgeführten Omnibus-Test ist abzulesen, dass Unterschiede innerhalb der einzelnen Kriterien bestehen. Für eine bessere Diskriminierung wurde ergänzend je Gruppe ein Post-hoc-Test durchgeführt. Tab. 1 gibt einen Überblick über die refraktiven Daten aller Augen im zeitlichen Verlauf.

### Einzelanalysen der Gruppen

In der Tab. 2 ist eine Auswahl der refraktiven Daten der 31 Patienten, die eine Blepharoplastik erhielten, im zeitlichen Verlauf gezeigt. Es zeigen sich im zeitlichen Verlauf keine signifikanten Veränderungen (*p* > 0,05).

**Table 1:** 

Refraktive Daten im Zeitverlauf. Alle Augen					
	*n*	Präoperativ	10 Tage postoperativ	3 Monate postoperativ	*p*-Wert *
Sphäre (D)	78	0,24 (±1,15)	0,44 (±0,99)	0,25 (±1,07)	0,164
Zylinder (D)	78	−0,81 (±0,31)	−0,91 (±0,40)	−0,86 (±0,41)	0,244
Sphärisches Äquivalent (D)	78	−0,23 (±0,98)	−0,15 (±0,91)	−0,20 (±1,02)	0,220
Visus	78	0,84 (±0,15)	0,79 (±0,19)	0,81 (±0,20)	0,139
Zentrale Pachymetrie	78	562,58 (±19,75)	561,91 (±20,74)	563,12 (±20,89)	0,163
K1	78	43,93 (±0,73)	43,85 (±0,77)	43,94 (±0,76)	0,246
K2	78	44,66 (±0,78)	44,59 (±0,90)	44,69 (±0,83)	0,71
Km	78	44,26 (±0,72)	44,24 (±0,82)	44,32 (±0,78)	0,629

* Einweg-ANOVA für verbundene winsorisierte Mittel

**Table 2:** 

	*n*	Präoperativ	10 Tage postoperativ	3 Monate postoperativ	*p*-Wert *
Sphäre (D)	31	0,15 (±0,90)	0,20 (±1,04)	0,24 (±0,90)	0,501
Zylinder (D)	31	−0,88 (±0,41)	−0,87 (±0,41)	−0,92 (±0,52)	0,945
Sphärisches Äquivalent (D)	31	−0,35 (±0,79)	−0,31 (±0,93)	−0,25 (±0,92)	0,481
Visus	31	0,93 (±0,10)	0,90 (±0,10)	0,93 (±0,09)	0,212
Zentrale Pachymetrie	31	567,42 (±15,39)	569,48 (±12,82)	569,10 (±18,02)	0,149
K1	31	43,45 (±0,75)	43,42 (±0,71)	43,46 (±0,73)	0,111
K2	31	44,25 (±0,90)	44,24 (±0,95)	44,25 (±0,96)	0,695
Km	31	43,89 (±0,73)	43,91 (±0,77)	43,93 (±0,82)	0,384

* Einweg-ANOVA für verbundene winsorisierte Mittel

Es besteht zum Zeitpunkt des Fadenzugs nach 10 Tagen keine Visusreduktion, welche ggf. aus persistierender Schwellung hätte resultieren können, mehr.

Die Sphäre reichte in dieser Gruppe von −3,75 bis 2,25 dpt und der Zylinder von −3,00 bis 0,00.

**Figure 1:**
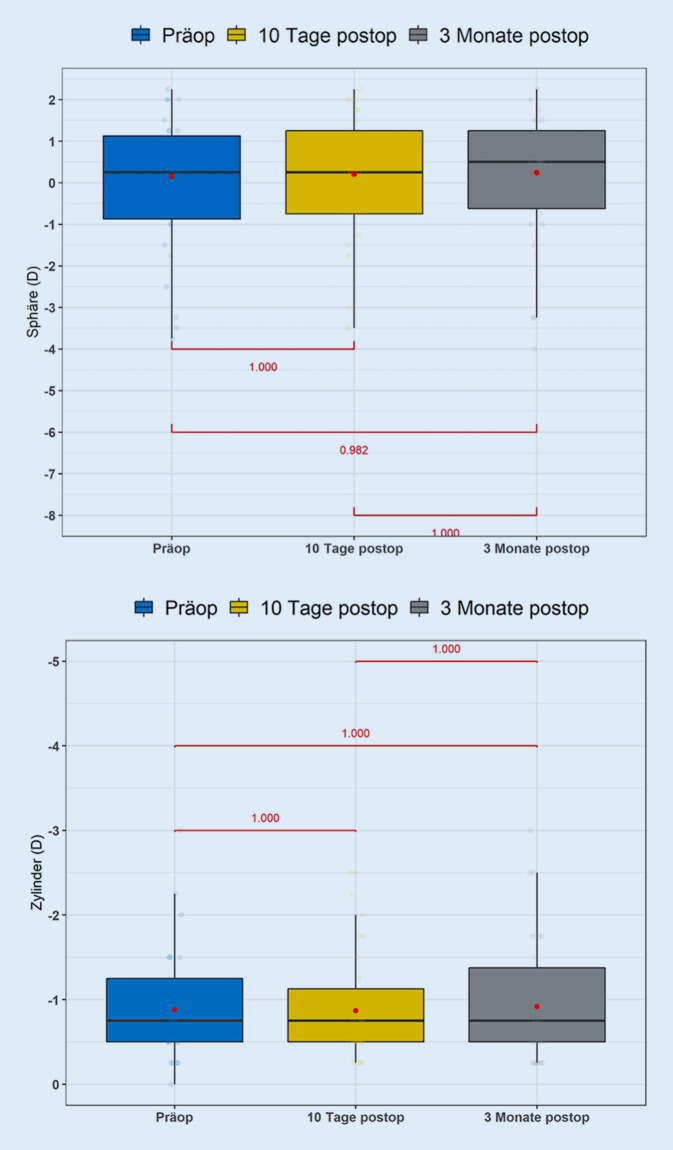

Auch in der Abb. 1 in der Darstellung in Boxplots lässt sich neben der Verteilung der refraktiven Daten dieser Gruppe entnehmen, dass sich keine signifikante Veränderung der Daten zeigt.

28 Patienten absolvierten in unserer Studie nach der Levatorresektion das Follow- up vollständig (Tab. 3). Nach 10 Tagen bestand eine geringfügige, wenn auch statistisch nicht signifikante, Visusreduktion, welche sich ggf. durch die postoperative Schwellung erklären lässt.

**Table 3:** 

Refraktive Daten im Zeitverlauf. Levatorresektion					
	*n*	Präoperativ	10 Tage postoperativ	3 Monate postoperativ	*P*-Value*
Sphäre (D)	28	0,66 (±1,51)	0,81 (±1,00)	0,54 (±1,51)	0,36
Zylinder (D)	28	−0,72 (±0,23)	−0,91 (±0,47)	−0,71 (±0,38)	0,041
Sphärisches Äquivalent (D)	28	0,23 (±1,51)	0,35 (±1,05)	0,18 (±1,44)	0,737
Visus	28	0,86 (±0,15)	0,78 (±0,19)	0,81 (±0,20)	0,271
Zentrale Pachymetrie	28	557,04 (±20,23)	558,11 (±21,51)	559,86 (±22,47)	0,584
K1	28	44,42 (±0,64)	44,30 (±0,72)	44,46 (±0,66)	0,343
K2	28	45,01 (±0,40)	45,00 (±0,56)	45,08 (±0,46)	0,805
Km	28	44,65 (±0,51)	44,69 (±0,57)	44,72 (±0,62)	0,778

* Einweg-ANOVA für verbundene winsorisierte Mittel

Es zeigte sich jedoch eine temporäre, signifikante Veränderung des Zylinders von präoperativ zum Zeitpunkt des Fadenzugs. Die Achslage zeigte sich hierbei nicht signifikant verändert.

Zur Abschlusskontrolle 3 Monate postoperativ normalisierte sich der Zylinder auf das präoperative Niveau.

Der Wilcoxon-Vorzeichen-Test, adjustiert mit der Bonferroni-Methode, zeigte einen signifikanten Anstieg der Zylinderwerte 10 Tage postoperativ (*p* = 0,042).

**Figure 2:**
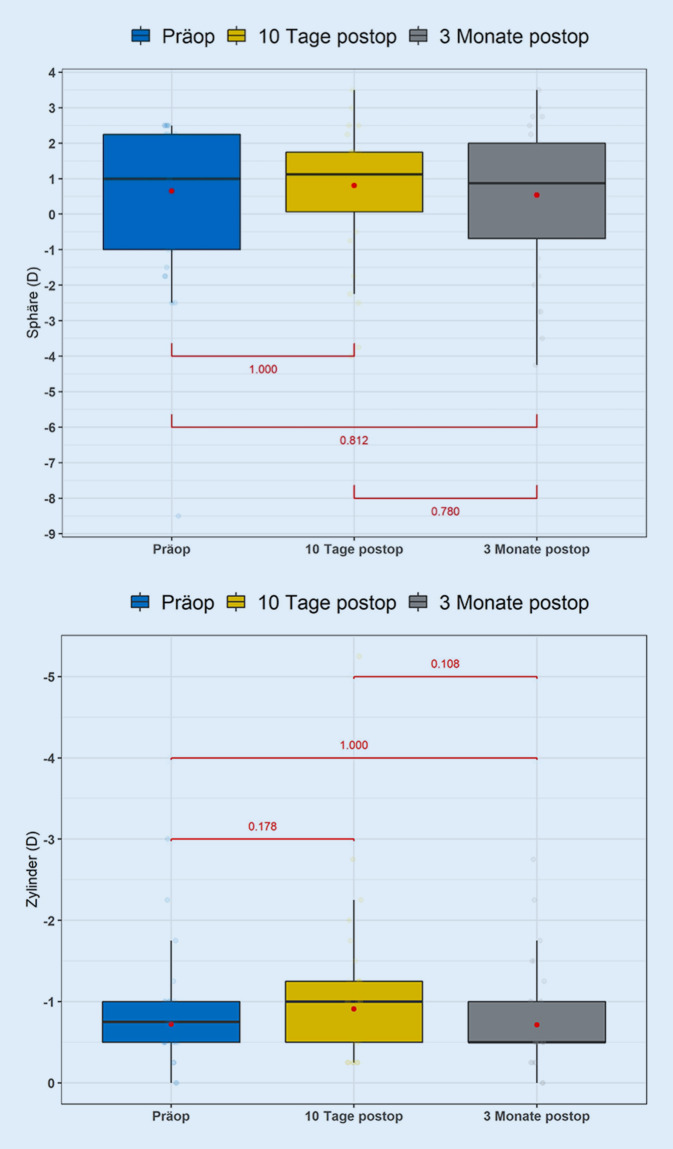

Trotz des signifikanten Omnibus-Tests bei den Zylinderwerten zeigten sich keine signifikanten Unterschiede bei paarweisen Vergleichen der getrimmten Mittel, wenn die *p*-Werte für multiples Testen korrigiert wurden. Man kann also eine Tendenz in der Zylinderveränderung beobachten, die jedoch noch mit mehr Fällen untersucht werden sollte (Abb. 2).

In der Tab. 4 finden sich die refraktiven Daten der 19 Patienten mit lateraler Zügelplastik aufgelistet. Es bestehen im gesamten zeitlichen Verlauf keine signifikanten Veränderungen.

**Table 4:** 

	*n*	Präoperativ	10 Tage postoperativ	3 Monate postoperativ	*p*-Wert*
Sphäre (D)	19	−0,24 (±0,85)	−0,12 (±0,59)	−0,13 (±1,08)	0,838
Zylinder (D)	19	−0,99 (±0,49)	−1,00 (±0,58)	−1,04 (±0,58)	0,940
Sphärisches Äquivalent (D)	19	−0,72 (±0,69)	−0,62 (±0,66)	−0,61 (±0,82)	0,453
Visus	19	0,64 (±0,17)	0,63 (±0,19)	0,71 (±0,19)	0,636
Zentrale Pachymetrie	19	562,11 (±26,34)	557,89 (±24,59)	559,16 (±21,48)	0,524
K1	19	43,96 (±0,87)	43,97 (±0,87)	43,97 (±0,80)	0,81
K2	19	44,51 (±1,44)	44,27 (±1,47)	44,42 (±1,23)	0,442
Km	19	44,13 (±1,20)	44,06 (±1,31)	44,03 (±1,22)	0,754

Ursprünglich war eine vierte Gruppe geplant, welche die Entropiumkorrekturen nach Quickert oder Wies umfassen sollte. Es wurden im gesamten Studienzeitraum wegen der genannten Ausschlusskriterien lediglich 3 Patienten eingeschlossen, jedoch absolvierte nur einer das Follow-up vollständig. Somit wurde diese Gruppe vor der Auswertung separiert und nicht mehr analysiert.

**Figure 3:**
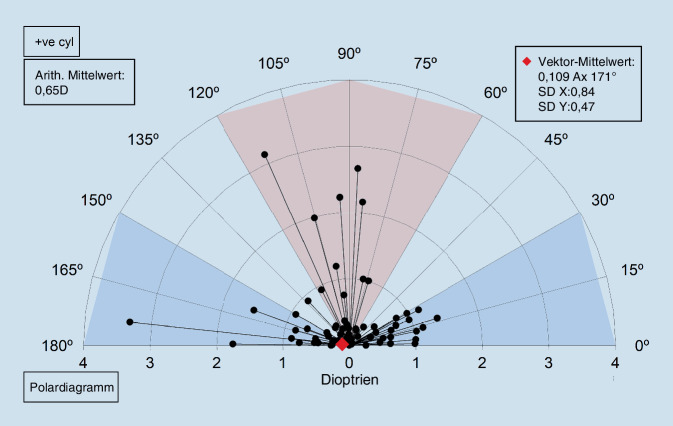

**Figure 4:**
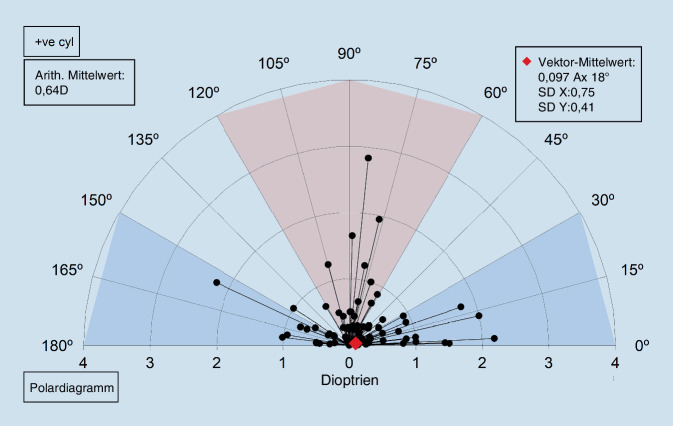

In der Abb. 3 zeigt sich der chirurgisch induzierte Astigmatismus 10 Tage postoperativ, und in der Abb. 4 sind die entsprechenden Daten nach 3 Monaten grafisch dargestellt.

Da bei den chirurgischen Prozeduren in diesem Fall keine Refraktionsänderung durch die Operation angestrebt wurde, ist der zielinduzierte Astigmatismusvektor (TIA) mit 0 anzunehmen.

Es zeigt sich, dass es einen Unterschied von prä- zu postoperativ gibt (arithm. Mittel liegt bei 0,6 dpt), jedoch existiert kein klarer vektorieller Trend (mittlerer Vektor nahe 0).

## Diskussion

Fehlstellungen der Augenlider, wie z. B. Ptosis oder Blepharochalasis, können die corneale Oberfläche modifizieren und ggf. auch refraktive Fehler induzieren [4]. Gerade bei Kindern gewinnt dieser Umstand an besonderer Bedeutung durch die ggf. resultierenden Einschränkungen der Visusentwicklung, die zur Entstehung einer Amblyopie (=Schwachsichtigkeit) führen kann.

Es sollte jedoch der Effekt, den eine veränderte Zugrichtung der Augenlider oder auch postoperative Gewebeschwellungen auf die corneale Topographie haben kann, nicht vernachlässigt werden. Hierbei stehen Veränderungen v. a. des Astigmatismus im Vordergrund. Holck et al. haben bereits 1998 22 Patienten, welche sich einer Levatorresektion bzw. -refixation unterziehen mussten, analysiert.

Die Patienten wurden jeweils präoperativ sowie nach 6 Wochen postoperativ mit der EyeSys-Topographie (EyeSys, Houston, TX, USA) untersucht. Lediglich ein kleiner Teil der Patienten (*n* = 10) wurden im Rahmen einer Abschlusskontrolle nach 12 Monaten erneut untersucht.

Sie konnten eine Steigerung des Astigmatismus in 86 % der Fälle zeigen [5]. Der erworbene Astigmatismus schien in ihrer Versuchspopulation rückläufig zu sein. In dieser Studie wurde der Visus und auch die Refraktion nicht mit betrachtet.

In unserer Patientengruppe zeigte sich eine temporäre Veränderung des Zylinders von −0,72 ± 0,23 dpt (präoperativ) auf −0,91 ± 0,47 dpt (10 Tage postoperativ), welche mit *p* = 0,042 auch statistisch signifikant ist.

Nach der abgeschlossenen Wundheilung nach 3 Monaten ist im Vergleich zum präoperativen Niveau keine signifikante Veränderung mehr festzustellen.

Savino et al. analysierten 20 Augen, behandelt mit einer Ptosisoperation bei entweder erworbener oder angeborener Ptosis. Durch die Korrektur der Lidfehlstellung kam es zu einer Wiederherstellung der Regularität der anterioren Kurvatur der cornealen Topographie und der cornealen Symmetrie [4]. Sie sahen in ihrem Patientenkollektiv eine superiore Aufsteilung in der Topographie, welche sich jedoch nach der Ptosiskorrektur normalisierte [4].

In einer Arbeit von Cadrea et al. 1992 wurden 88 Patienten verglichen, welche sich einer operativen Ptosiskorrektur unterzogen hatten [6]. Die Kontrollpunkte in dieser Studie waren nach 3, 6 und 12 Monaten postoperativ.

29 der Patienten bekamen eine Levatorresektion und zeigte dabei einen höheren Anstieg des Zylinders über den gesamten Zeitraum (Anstieg von 0,50 dpt) als die Patienten, welche eine Frontalissuspension erhalten hatten (Anstieg von 0,20 dpt) [6].

Im Jahr 1995 untersuchten Gingold et al. 47 Augen von 26 Patienten präoperativ, sowie 6 Monate nach Ptosischirurgie. Sie konstatierten zwar, dass die Patienten subjektive Veränderungen bemerkten, jedoch sich keine signifikanten Veränderungen hinsichtlich Sphäre, Zylinder, Achslage oder Keratometrie zeigten [10].

Zinkernagel et al. untersuchten 43 Patienten, welche entweder eine Ptosiskorrektur oder Blepharoplastik erhalten hatten [7]. Sie konnten zeigen, dass nach der Ptosisoperation der Astigmatismus signifikant gesunken ist (0,25 dpt; *p* = 0,02). Ebenso zeigte sich eine signifikante Veränderung nach Blepharoplastik mit Reduktion ausgeprägter Fettpolster (0,21 dpt; *p* = 0,04). Bei Blepharoplastiken, bei denen intraoperativ lediglich der Hautüberschuss entfernt werden musste, zeigte sich eine deutlich geringere Veränderung im Astigmatismus (0,09 dpt) [7].

Simsek et al. veröffentlichten im Jahr 2015 eine Auswertung von 43 Augen von 23 Patienten, bei denen vor der Blepharoplastik sowie postoperativ nach 1 und 3 Monaten eine Pentacam durchgeführt wurde [8]. Sie konnten eine signifikante Veränderung bzw. Erhöhung des Astigmatismus an beiden postoperativen Zeitpunkten im Vergleich zu präoperativ zeigen. Es zeigte sich eine mittlere Veränderung des Astigmatismus von 0,15 D [8].

Die Achslage des Astigmatismus zeigte keine signifikante Veränderung [8].

In unserer Studienpopulation erfolgte eine Kontrolle nach 10 Tagen, zum Zeitpunkt des Fadenzugs und nach 3 Monaten. Wir konnten im Gegensatz zu der vorgelegten Arbeit von Simsek et al. bei ähnlich großer Patientenzahl keine signifikante Veränderung des Astigmatismus nach Blepharoplastik nachweisen. Bei unseren 31 Patienten bestand präoperativ ein Astigmatismus von −0,88 ± 0,41 dpt, nach 10 Tagen −0,87 ± 0,41 dpt und nach 3 Monaten von −0,92 ± 0,52 dpt. Somit zeigt sich auch in unserer Studiengruppe nach der Blepharoplastik eine Erhöhung des Astigmatismus, jedoch ohne statistische Signifikanz.

Ekin et al. untersuchten in 2019 in ihrer Studie 103 Patienten prospektiv hinsichtlich einer Veränderung ihres Visus und visuellen Funktionen wie beispielsweise Kontrastsensitivität vor und nach Oberlidblepharoplastik [9].

Sie konnten keine signifikante Veränderung des Visus (*p* = 0,157) präoperativ zu den postoperativen Werten nach einem Monat feststellen [9]. Das Kontrastsehen hat sich in diesem Zeitraum signifikant verbessert.

Im Gegensatz zu der Studie von Simsek et al. und auch unseren Ergebnissen konnten sie eine signifikante Verringerung des Astigmatismus feststellen.

Die Vorteile unserer Studie liegen unseres Erachtens darin, dass verschiedene Operationsverfahren im gleichen Setting betreut und verglichen werden können. Gleichzeitig konnten die Patienten nach einer dreimonatigen Nachbeobachtungszeit untersucht werden. Ihre Datensätze flossen in die Auswertung ein, wir konnten zu jedem der Kontrollzeitpunkte auch eine Topographie durchführen.

Auffällig ist, dass wir lediglich 19 Datensätze von Patienten mit lateraler Zügelplastik zur Analyse zur Verfügung hatten, gemessen an der hohen Zahl, die im Zeitraum operiert wurde. Dies lässt sich zum einen dadurch erklären, dass viele dieser Patienten ambulant operiert wurden und die Nachsorge, beginnend bei der Kontrolle am ersten postoperativen Tag bis zum Fadenzug, bereits mit dem Hausaugenarzt geplant hatten.

Zum anderen handelt es sich hier um eine vergleichsweise kleine operative Prozedur, bei der die Patienten aufgrund des komplikationslosen Verlaufs teils – gerade bei weiten Anreisen – schwer zu motivieren waren, zu einer Abschlusskontrolle nach 3 Monaten zu kommen.

Als Limitation unserer Studie ist klar anzuführen, dass die Ergebnisse lediglich für die hier angeführten operativen Prozeduren zu verwerten ist.

Zusammenfassend lässt sich konstatieren, dass sich in unserem Patientenkollektiv eine temporäre signifikante Veränderung des Astigmatismus lediglich nach Levatorresektion nachweisen ließ. Weder bei der Blepharoplastik noch bei der lateralen Zügelplastik konnten wir signifikante Veränderungen nachweisen.

Im Zuge der Patientenaufklärung sollten die Patienten auch auf die Möglichkeit einer, zumindest nach derzeitiger Studienlage, temporären Veränderung des Astigmatismus hingewiesen werden. Gegebenenfalls kann es sinnvoll sein, bei subjektiv empfundener Visusverschlechterung die Refraktion und auch die Topographie postoperativ zu überprüfen und einen refraktiven Ausgleich vorzunehmen.

## Fazit für die Praxis

- Eine sorgfältige und umfassende Patientenaufklärung ist vor jeder operativen Prozedur essenziell und gut zu dokumentieren.
- Vor lidchirurgischen Eingriffen sollte auf die Möglichkeit einer – nach derzeitiger Studienlage – temporären Refraktionsänderung hingewiesen werden.
- Postoperativ sollte bei Visusminderung die Refraktion und auch die Topographie überprüft werden.
